# Reduced plasma level of basic fibroblast growth factor is associated with incomplete device endothelialization at six months following left atrial appendage closure

**DOI:** 10.1186/s12872-021-02059-6

**Published:** 2021-05-16

**Authors:** Jing Xu, Xin Gong, Chuanzhi Chen, Jun Xing, Qi Wang, Weifeng Shen, Qi Zhang

**Affiliations:** 1 Department of Cardiology, Shanghai East Hospital, Shanghai, Tongji University School of Medicine, No. 150 Ji Mo Rd, Shanghai, 200120 People’s Republic of China; 2 Department of Heart Failure, Shanghai East Hospital, Tongji University School of Medicine, Shanghai, People’s Republic of China; 3 Department of Radiology, Shanghai East Hospital, Tongji University School of Medicine, Shanghai, People’s Republic of China; 4 Department of Ultrasonography, Shanghai East Hospital, Tongji University School of Medicine, Shanghai, People’s Republic of China; 5 Department of Cardiology, Ruijin Hospital, Shanghai Jiao Tong University School of Medicine, Shanghai, 200025 People’s Republic of China

**Keywords:** Left atrial appendage closure, Basic fibroblast growth factor, Biomarker, Device-related endothelialization

## Abstract

**Objectives:**

To investigate whether inflammatory and growth factors (IGFs) were associated with incomplete device endothelialization (IDE) at 6 months after successful left atrial appendage closure (LAAC).

**Background:**

IDE after LAAC is correlated with device-related thrombus (DRT) formation and subsequent thromboembolic events. However, biomarkers for early detection of IDE remain lacking.

**Methods:**

Plasma levels of IGFs including basic fibroblast growth factor (bFGF), platelet derived growth factor (PDGF), stromal cell derived factor (SDF)-1a, transforming growth factor (TGF)-β_1_, vascular growth factor receptor-1 (VEGF-R_1_) and von Willebrand factor (vWF) were determined using ELISA kits in 55 consecutive patients with atrial fibrillation (AF) at 6 months after LAAC with Watchman devices. The status of device endothelialization was assessed by transesophageal echocardiography and cardiac CT.

**Results:**

IDE and complete device endothelialization(CDE)were detected in 38 and 17 patients, respectively. Among the six IGFs, only plasma level of bFGF was significantly lower in patients with IDE compared to those with CDE (303.49 ± 246.84 vs. 556.31 ± 197.84 pg/ml, *p* < 0.001). C-statistics of plasma bFGF for discriminating patients with IDE from those with CDE was 0.785 (95 % CI: 0.663–0.907, *p* < 0.001), with a cut-off value of 440.52pg/ml (sensitivity 0.765; specificity 0.789). Multivariate logistic regression model showed that lower bFGF was an independent factor for IDE (OR: 11.752, 95 % CI: 2.869–48.144, *P* = 0.001). bFGF improved the classification of patients (NRI: 0.677,95 % CI: 0.320–1.033, *p* = 0.004).

**Conclusions:**

Reduced plasma bFGF level confers an increased risk for IDE after LAAC. Further prospective studies are warranted to examine if bFGF could serve as a biomarker for IDE post LAAC.

## Introduction

Left atrial appendage closure (LAAC) is recommended by the latest guidelines for stroke prevention in atrial fibrillation (AF) patients [1, 2]. At present, duration of postprocedural antithrombotic therapy is mainly based on animal studies, showing that complete device endothelialization (CDE) is achieved at 3 months after LAAC [3]. Nevertheless, incomplete device endothelialization (IDE) often occurs in humans, which could result in device-related thrombus (DRT) formation and subsequent thromboembolic clinical events [4–7]. Thus, early detection of IDE is mandatory for adjusting antithrombotic strategies to prevent DRT formation. Currently, transesophageal echocardiography (TEE) and cardiac CT are clinically used for assessing device endothelialization following LAAC [8–12]. However, some individuals are intolerant to TEE and cardiac CT could increase the risk of contrast-induced nephropathy for patients with renal dysfunction, highlighting that certain easily accessible and clinically applicable blood biomarkers for diagnosing IDE after LAAC are strongly desirable.

Inflammatory and growth factors (IGFs) such as basic fibroblast growth factor (bFGF), platelet derived growth factor (PDGF), stromal cell derived factor (SDF)-1a, transforming growth factor (TGF) -β_1_, vascular growth factor receptor-1 (VEGF-R_1_) and von Willebrand factor (vWF) have been proved to regulate a variety of biological functions during wound healing process [13, 14]. In humans, IGFs have been proposed as biomarkers for device neo-endothelialization after percutaneous closure of atrial septal defect [15]. Meanwhile, in early endothelialization of cardiovascular devices including prosthetic heart valves and vascular stents, a set of biological cascades promote platelet adhesion and release of bioactive IGFs, which induce smooth muscle cell migration and proliferation [16]. Schwartz et al. have observed a similar healing process after LAAC in canine model [3], suggesting that IGFs may also play a role in the device endothelialization after LAAC. However, the association between IGFs and LAAC device endothelialization in clinical practice has not yet been reported. In this study, we sought to investigate whether IGFs could serve as a biomarker for IDE after successful LAAC.

## Methods

### Study population and design

**Fig. 1:**
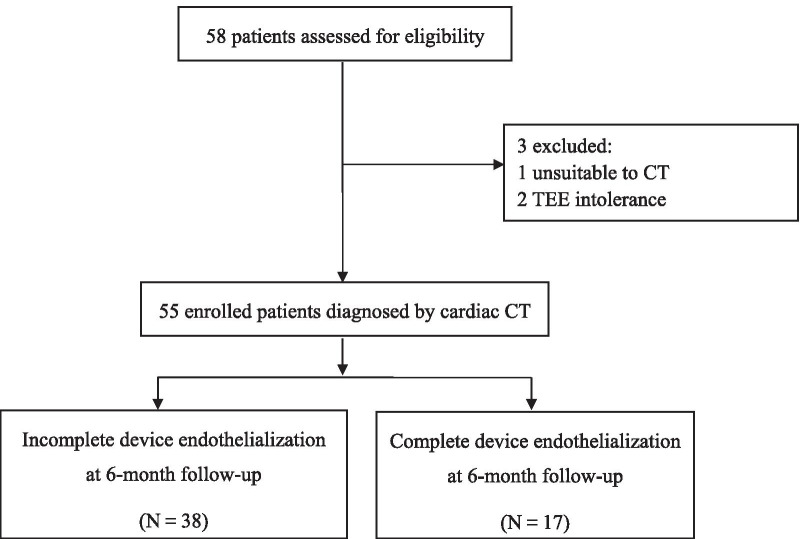
Flowchart of patient selection

A total of 58 consecutive patients with AF undergoing successful percutaneous LAAC from March to December 2019 at Shanghai East Hospital, Tongji University, China, were enrolled. Three patients were excluded because of unsuitable for cardiac CT due to renal failure (n = 1) or intolerance to TEE (n = 2). Thus, the remaining 55 patients who had complete 6-month clinical follow-up with TEE and cardiac CT post LAAC were eligible for final analysis. Among them, 23 had paroxysmal AF and 32 patients were in persistent or permanent AF. Based on the quantitative results of cardiac CT, IDE and CDE were detected in 38 (69 %) and 17 (31 %) patients, respectively (Fig. 1).

This study was carried out according to the principles of the declaration of Helsinki and was approved by the hospital ethics committee. All patients provided their written informed consents.

### LAAC procedure and medical treatment

LAAC was performed according to standardized protocol. Briefly, the procedure was done under conscious sedation, and guided by angiography and TEE in all cases. The size of the device was selected based on angiographic and echo measurements, including LAA orifice, diameter, depth, and morphology [17].

All patients received anticoagulation (warfarin or non-vitamin K antagonist oral anticoagulants) in the initial 45 days post-procedure [18]. For those who underwent one-stage LAAC and catheter ablation for AF, anticoagulation was prescribed following the procedure for at least 3 months [19]. If there was no DRT or peri-device leak of greater than 5 mm at 45-day or 3-month follow-up, anticoagulation was discontinued and dual antiplatelet therapy with aspirin (100 mg) and clopidogrel (75 mg) daily was initiated until 6 months. Beta-blocker, angiotensin-converting-enzyme inhibitor or angiotensin II-receptor blocker, calcium channel blocker, and statins were given according to the guideline recommendation and patient’s individual risk factors [20, 21].

### Definitions of IDE and assessment of device endothelialization

**Fig. 2:**
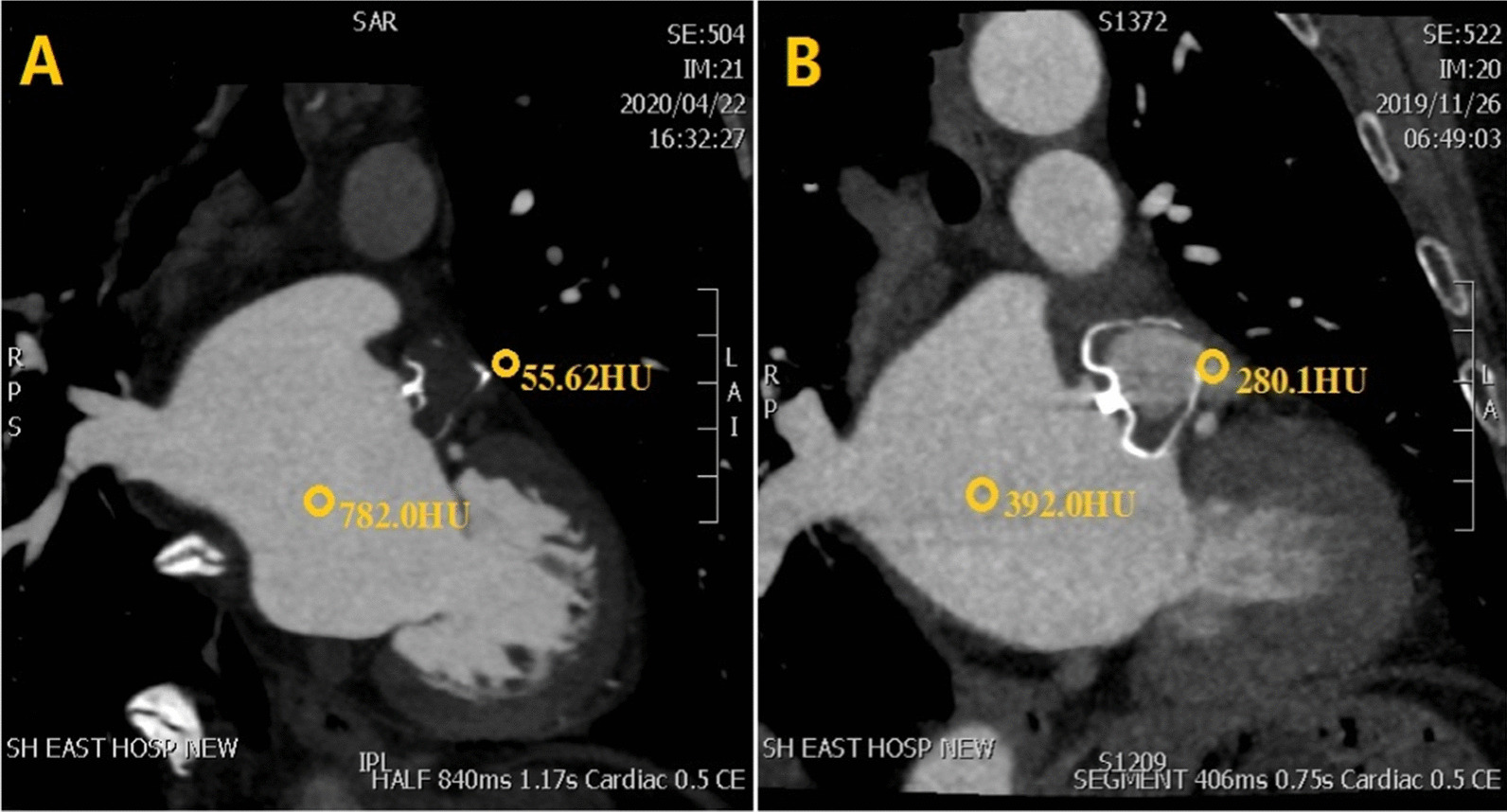
Watchman device endothelialization assessed by cardiac CT. **a** complete occlusion of the left atrial appendage (no trans-fabric leak, LAA HU < 100 and LA/LAA < 0.25). **b** Incomplete endothelial coverage of the device (trans-fabric leak, LAA HU > 100)

The degree of left atrial appendage (LAA) occlusion and type of leak were assessed by cardiac CT and TEE. IDE was defined as LAA attenuation > 100 Hounsfield unit (HU) or LAA to left atrium attenuation ratio ≥ 0.25, and the presence of trans-fabric leak on cardiac CT [11] (Fig. 2).

#### Cardiac CT

ECG-gated (systolic-triggered) cardiac CT was performed using a 320-detector row CT system (Aquilion ONE™, Toshiba Medical Systems Corporation, Tokyo, Japan) with a collimation of 320 × 0.5mm. Tube current was adapted automatically according to body mass index (BMI) (100 kV for BMI < 30; 120 kV for BMI > 30). Each patient received an injection of 50 to 70 mL of contrast medium (Iohexol, 350 mg iodine/mL) at 4.0 to 5.0 mL/s. For the purpose of this study, a double-phase protocol was applied. After first-pass imaging, the second set of images were acquired for 30 s to assess delayed contrast opacification. Images of all scans were reconstructed to generate 0.5mm slice thickness images with a 0.25mm increment and a medium sharp convolution kernel (FC47).

Image analysis was made using Vitrea Workstation™ (Vital, a Toshiba Medical Systems Group Company, Zoetermeer, Netherlands) by two blinded and experienced radiologists. LAA patency was assessed by HU measurements in the LAA distal to the device and comparison of contrast density between LAA and left atrium. Trans-fabric leak was defined as contrast entering into the LAA through the fabric rather than around the device.

#### TEE imaging

Two-dimensional TEE was performed with a Philips Epiq7C machine (Phillips, IE). LAA imaging was acquired in 0°, 45°, 90°, and 135° views at the mid-esophageal level. All images were analyzed by a trained echocardiographer who was blinded to baseline clinical information of the patients and results of cardiac CT. Peri-device leak was defined as a peri-device flow by color Doppler, and DRT was determined on the luminal side of the device. Endothelial coverage of each device was further assessed by three-dimensional color-flow Doppler.

### Biochemical assays

Blood samples for measuring IGFs were obtained in EDTA coated tubes and centrifuged for 15 min at 3000 rpm within 20 min of collection. Plasma samples were then stored at -80 °C for ELISA. Commercially available sandwich ELISA kits for the inactive form of bFGF, PDGF, SDF-1a, TGF-β_1_, VEGF-R_1_ and vWF (Weiao Biological Company, Shanghai, China) were used, following the manufacturers’ instructions. The absorbance of each well was measured with a microtiter plate reader (DENLEY DRAGON Wellscan MK 3, Thermo, Finland).

### Statistical analysis

Continuous variables are presented as mean ± SD. Normally distributed variables were compared using independent t test while Mann-Whitney U test used for non-normal distribution variables. Categorical data are summarized as frequencies and proportions, and Chi-square test was used to compare between the two subgroups. Logistic regression analysis was used to evaluate risk factors for IDE. Receiver operating characteristic (ROC) analysis and the calculation of the area under the curve (AUC) were performed to examine the efficiency of distinguishing IDE patients from those with CDE. C-statistics with 95 % confidence interval (CI) were reported. Continuous net reclassification improvement (NRI) index was calculated by R 4.0.2 to assess the added value of bFGF in reclassification of the patients compared with TEE. Other analyses were done by the statistics software SPSS (SPSS Inc, version 22.0, Chicago, IL, USA). *P*-value of < 0.05 was considered statistically significant.

## Results

### Baseline characteristics

Patients with IDE had lower BMI than those with CDE (25.12 ± 3.17 vs. 27.85 ± 4.32 kg/m^2^, *p* < 0.05), but there was no significant difference in terms of age, gender, disease history, persistent/permanent AF, echocardiographic measurement and procedural characteristics between the two groups **(**Table 1**).**

**Table 1:** Baseline clinical characteristics

	Total population (n=55)	IDE (n = 38)	CDE (n = 17)	*P* value
Age (years)	70.71 ± 8.04	72.21 ± 8.141	69.56 ± 7.938	0.492
Male gender, n (%)	29 (52.7)	19 (50.0)	10 (58.8)	0.545
BMI (kg/m^2^)	25.97 ± 3.74	25.12 ± 3.17	27.85 ± 4.32	0.028
Hypertension, n (%)	44 (80.0)	31 (81.6)	13 (76.5)	0.662
Stroke, n (%), n (%)	23 (41.8)	16 (42.1)	7 (41.2)	0.949
Diabetes, n (%)	14 (25.5)	11 (28.9)	3 (17.6)	0.374
Coronary heart disease, n (%)	20 (36.4)	12 (31.6)	8 (47.1)	0.270
Persistent/permanent AF, n (%)	32 (58.2)	20 (52.6)	12 (70.6)	0.212
CHA_2_DS_2_-VASc score	4.45 ± 1.71	4.55 ± 1.639	4.24 ± 1.888	0.554
HAS-BLED score	2.67 ± 0.94	2.68 ± 0.933	2.65 ± 0.996	0.894
TTE measurement				
LA (mm)	44.65 ± 6.28	44.68 ± 4.662	44.59 ± 9.111	0.968
LVEF (%)	61.18 ± 6.79	60.74 ± 7.325	62.18 ± 5.457	0.423
LVEDD (mm)	47.35 ± 5.32	47.26 ± 5.51	47.53 ± 5.04	0.861
LVESD (mm)	31.45 ± 5.07	31.61 ± 5.38	31.12 ± 4.44	0.727
Procedural characteristics of LAAC				
Device size (mm)	26.82 ± 3.4	26.61 ± 3.33	27.29 ± 3.62	0.509
Device compression ratio (%)	21.62 ± 4.68	21.66 ± 4.46	21.54 ± 5.40	0.945
Peri-device leak (mm)	0.03 ± 0.23	0.04 ± 0.28	0.00 ± 0.00	0.324
Warfarin at discharge	19 (34.5)	13 (34.2)	6 (35.3)	0.938

BMI, body mass index; AF, atrial fibrillation; TTE, transthoracic echocardiography; LA, left atrium; LVEF, left ventricular ejection fraction; LVEDD, Left ventricular end diastolic diameter; LVESD, left ventricular end systolic diameter

### Plasma levels of IGFs

**Fig. 3:**
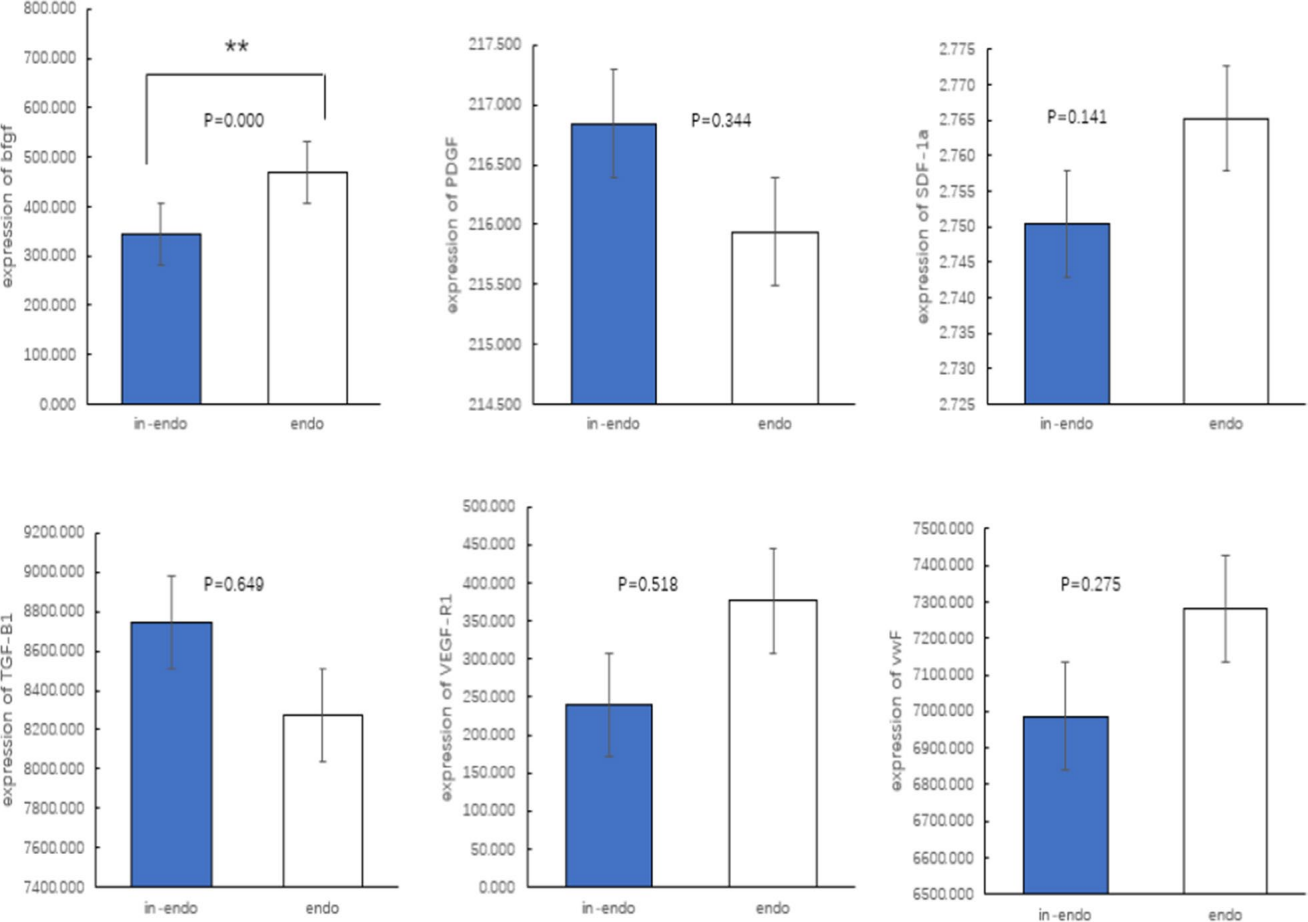
Plasma levels of bFGF (pg/ml), PDGF (pg/ml), SDF-1a (ng/ml), TGF-β_1_ (pg/ml), VEGF-R_1_ (pg/ml) and vWF (mu/ml) in patients with incomplete (blue bar) and complete device endothelialization (white bar). (**means *p* < 0.05 )

Level of bFGF in plasma was significantly lower in patients with IDE than that in patients with CDE (303.49 ± 246.84 vs. 556.31 ± 197.84 pg/ml, *p* < 0.001), whereas VEGF-R_1_, vWF, SDF-1a, PDGF and TGF-β_1_ levels were similar between the two groups (Fig. 3).

### Performance of plasma bFGF for discrimination of IDE

**Fig. 4:**
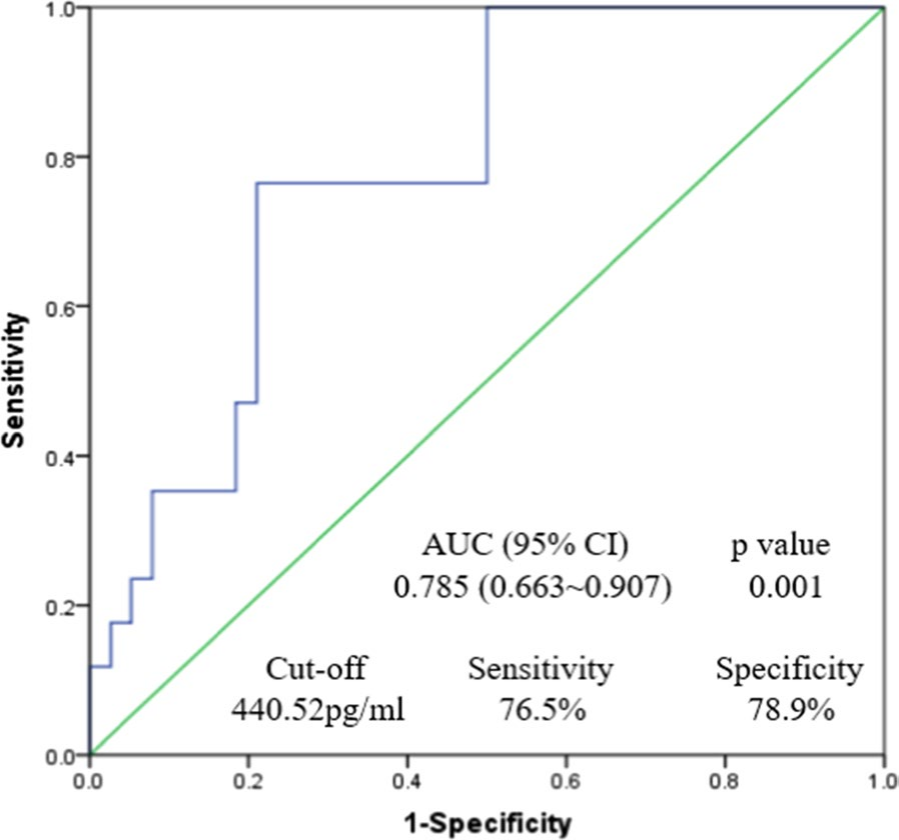
ROC curve analysis using plasma bFGF to distinguish incomplete device endothelialization

ROC analysis was performed to evaluate the diagnostic ability of bFGF for IDE. Plasma bFGF held a C-statistics of 0.785 (95 % CI: 0.663–0.907, *p* < 0.001), with a cut-off value of 440.52 pg/ml (sensitivity 76.5 %, specificity 78.9 %) (Fig. 4).

In univariate analysis, IDE was associated with BMI (OR 4.982, 95 %CI 1.418–17.509, *p* = 0.009), and bFGF (OR 12.188, 95 % CI 3.111–47.738, *p* = 0.000). Multivariate logistic regression model including bFGF, BMI, left ventricular ejection fraction, history of diabetes and device size showed that lower level of bFGF was the only independent factor for IDE (OR: 11.752, 95 % CI: 2.869–48.144, *P* = 0.001) (Table 2). More importantly, plasma bFGF levels allowed a significant reclassification of the patients compared with TEE (NRI: 0.677, 95 % CI: 0.320–1.033, *p* = 0.004).

**Table 2:** Multivariate analysis

Variables	OR	95 %CI	*P*
bFGF	11.752	2.869–48.144	0.001
BMI	2.479	0.521–11.792	0.254
LVEF	2.293	0.423–12.438	0.336
Diabetes	0.485	0.083–2.850	0.424
Device size	0.916	0.741–1.133	0.420

bFGF, basic fibroblast growth factor; BMI, body mass index; LVEF, left ventricular ejection fraction

## Discussion

The results of this study show that reduced bFGF levels in plasma were associated with higher risk of IDE and may hold potential in IDE discrimination at 6 months post successful LAAC.

The exact timing of CDE after LAAC remains unclear but is likely to be variable. In canine models, complete endothelialization occurred at 3 months [3, 22], whereas in humans delayed device endothelialization often existed after LAAC. In a case of hereditary hemorrhagic teleangectasia, IDE was present at 10 months after Watchman implantation [23]. Sharma et al. reported two cases of IDE at 1.5 and 2 years post LAAC, respectively [6]. Mcivor discovered that a Watchman device was not endothelialized even at 3 years after the procedure [24]. In the present study, all patients were selected specially as they had successful LAAC and received combined cardiac CT and TEE to assess device endothelialization. Interestingly, IDE defined as trans-fabric leak on cardiac CT at 6 months post LAAC was detected in more than two-thirds of patients (69.0 %). These findings support a notion that delayed device endothelialization was common in humans, and oral anticoagulation regimen post LAAC recommended by guidelines may not provide sufficient time for adequate endothelialization.

Conventionally, TEE is recommended to evaluate device competency and complications during follow-up after LAAC [25], but it is not quantitative and, to certain extent, operator-dependent. Recently, cardiac CT becomes a valid non-invasive alternative to TEE and is increasingly used for post-procedural surveillance, especially to clarify the device endothelialization and residual leak into the LAA because of its superior spatial resolution, three-dimensional assessment and largely operator-independent nature [11, 12, 26]. However, this technique has several limitations including contrast use and radiation exposure, precluding its extensive clinical use particularly in patients with severe renal impairment [10]. Hence, the quest for new clinically applicable biomarkers is highly desirable. The major finding of this study is that plasma bFGF level was significantly lower in patients with IDE. Reduced plasma bFGF provided relatively high accuracy in predicting IDE and remained an independent risk factor for IDE after adjusting for multiple confounders. These findings suggest that plasma bFGF may serve as a potential marker for diagnosing IDE after LAAC. Additionally, the result of NRI suggests that determination of plasma bFGF levels could provide better information than TEE in terms of reclassification of patients into more appropriate diagnostic groups.

The process of neoendothelialization and cellular healing of LAAC device is likely to be complex, involving early thrombus organization, newly formed granulation tissue comprising fibroblasts, blood vessels, inflammatory cells and final healing by connective tissue [3, 22, 27]. The entire dynamic process depends, at least partly, on a series of IGFs. As one of the most important IGFs involved in wound healing, bFGF stimulates angiogenesis by promoting endothelial cell morphogenesis [14]. Previous studies have identified that bFGF attenuates myocardial ischemia- reperfusion injury via activation of two downstream signaling pathways, namely PI3K/Akt and ERK1/2 [28]. In view of that PI3K/Akt pathway is a classic one associated with a variety of cellular biological processes including wound healing [29, 30], it could be hypothesized that bFGF may promote cardiac device endothelialization through activation of PI3K/Akt pathway.

Our study has important clinical relevance. It is well-recognized that early detection of IDE could result in a better safety and effectiveness of LAAC as DRT formation due to IDE may cause incident ischemic events [5, 6, 23, 31–33]. Current practice on duration of anticoagulant therapy (i.e., 45 days with warfarin immediately after LAAC [34]) was mainly based on the findings from animal models, showing that atrial-facing surface of the occluder is largely endothelialized at 45 days following the procedure [3]. In this study, two patients with reduced bFGF level and IDE developed DRT detected by TEE and cardiac CT at 6 months post LAAC, which was dissolved with intense anticoagulant treatment, and no ischemic stroke occurred. This observation highlights that prolonged follow-up and anticoagulant therapy are encouraged particularly for patients with IDE and reduced plasma bFGF.

### Limitations

The current study has several limitations. First, the number of patients was limited and data were all derived from a single center, so that some kind of selection bias could not be ruled out. Second, plasma bFGF was determined on the second day of admission only, therefore, the relationship between change of bFGF levels and IDE after implantation remained unclear. Further studies with a large sample size and dynamic evaluation of bFGF are warranted to confirm the utility of plasma bFGF as a novel biomarker for IDE after LAAC.

## Conclusions

The current study indicates that plasma bFGF with a level less than 440.52pg/ml confers a high risk of IDE at 6 months after LAAC. These findings may provide novel insight in the management of patients with AF undergoing LAAC.

## Data Availability

The datasets used in the case are available from the corresponding author upon reasonable request.

## Abbreviations

IGFs: inflammatory and growth factors
IDE: incomplete device endothelialization
LAAC: left atrial appendage closure
DRT: device-related thrombus
bFGF: basic fibroblast growth factor
PDGF: platelet derived growth factor
SDF: stromal cell derived factor
TGF: transforming growth factor
VEGF-R_1_: vascular growth factor receptor-1
vWF: von Willebrand factor
CDE: complete device endothelialization
TEE: transesophageal echocardiography
HU: Hounsfield unit
LAA: left atrial appendage
BMI: body mass index
ROC: Receiver operating characteristic
AUC: area under the curve
CI: confidence interval
NRI: net reclassification improvement

## References

[1] Hindricks G, Potpara T, Dagres N, Arbelo E, Bax JJ, Blomström-Lundqvist C, Boriani G, Castella M, Dan GA, Dilaveris PE (2020). 2020 ESC Guidelines for the diagnosis and management of atrial fibrillation developed in collaboration with the European Association of Cardio-Thoracic Surgery (EACTS). Eur Heart J.

[2] January CT, Wann LS, Calkins H, Chen LY, Cigarroa JE, Cleveland JC, Ellinor PT, Ezekowitz MD, Field ME, Furie KL (2019). 2019 AHA/ACC/HRS focused update of the 2014 AHA/ACC/HRS guideline for the management of patients with atrial fibrillation: a report of the American College of Cardiology/American Heart Association Task Force on Clinical Practice Guidelines and the Heart Rhythm Society in Collaboration With the Society of Thoracic Surgeons. Circulation.

[3] Schwartz RS, Holmes DR, Van Tassel RA, Hauser R, Henry TD, Mooney M, Matthews R, Doshi S, Jones RM, Virmani R (2010). Left atrial appendage obliteration: mechanisms of healing and intracardiac integration. JACC Cardiovasc Interv.

[4] Lammers J, Elenbaas T, Meijer A (2013). Thrombus formation on an Amplatzer closure device after left atrial appendage closure. Eur Heart J.

[5] Prosperi-Porta G, Schnell G, Colbert J, Franko A, Wilton SB, Kuriachan VP (2018). Multiple thromboembolic events from a left atrial appendage occlusion device. Can J Cardiol.

[6] Sharma SP, Singh D, Nakamura D, Gopinathannair R, Lakkireddy D (2019). Incomplete endothelialization of WatchmanTM device: predictors and implications from two cases. J Atrial Fibrillation.

[7] Sinanis T (2019). A very late and persistent thrombosis after left atrial appendage occlusion. Heart Views: Off J Gulf Heart Assoc.

[8] Cochet H, Iriart X, Sridi S, Camaioni C, Corneloup O, Montaudon M, Laurent F, Selmi W, Renou P, Jalal Z (2018). Left atrial appendage patency and device-related thrombus after percutaneous left atrial appendage occlusion: a computed tomography study. Eur Heart J Cardiovasc Imaging.

[9] Jaguszewski M, Manes C, Puippe G, Salzberg S, Muller M, Falk V, Luscher T, Luft A, Alkadhi H, Landmesser U (2015). Cardiac CT and echocardiographic evaluation of peri-device flow after percutaneous left atrial appendage closure using the AMPLATZER cardiac plug device. Catheter Cardiovasc Interv.

[10] Qamar SR, Jalal S, Nicolaou S, Tsang M, Gilhofer T, Saw J (2019). Comparison of cardiac computed tomography angiography and transoesophageal echocardiography for device surveillance after left atrial appendage closure. EuroIntervention.

[11] Saw J, Fahmy P, DeJong P, Lempereur M, Spencer R, Tsang M, Gin K, Jue J, Mayo J, McLaughlin P (2015). Cardiac CT angiography for device surveillance after endovascular left atrial appendage closure. Eur Heart J Cardiovasc Imaging.

[12] Sivasambu B, Arbab-Zadeh A, Hays A, Calkins H, Berger RD (2019). Delayed endothelialization of watchman device identified with cardiac CT. J Cardiovasc Electrophysiol.

[13] Fernlund E, Gyllenhammar T, Jablonowski R, Carlsson M, Larsson A, Arnlov J, Liuba P (2017). Serum biomarkers of myocardial remodeling and coronary dysfunction in early stages of hypertrophic cardiomyopathy in the young. Pediatr Cardiol.

[14] Sinno H, Prakash S (2013). Complements and the wound healing cascade: an updated review. Plast Surg Int.

[15] Aydin Sahin D, Baspinar O, Sulu A, Karsligil T, Kul S (2017). A comparison of the in vivo neoendothelialization and wound healing processes of three atrial septal defect occluders used during childhood in a nonrandomized prospective trial. Anatol J Cardiol.

[16] Jana S (2019). Endothelialization of cardiovascular devices. Acta Biomater.

[17] Fastner C, Hoffmann L, Aboukoura M, Behnes M, Lang S, Borggrefe M, Akin I, Nienaber CA (2018). Real-world experience comparing two common left atrial appendage closure devices. BMC Cardiovasc Disord.

[18] Ajmal M, Hutchinson MD, Lee K, Indik JH (2021). Outcomes in patients implanted with a Watchman device in relation to choice of anticoagulation and indication for implant. J Interv Card Electrophysiol.

[19] Chen M, Wang ZQ, Wang QS, Sun J, Zhang PP, Feng XF, Li W, Yu Y, Liu B, Mo BF (2020). One-stop strategy for treatment of atrial fibrillation: feasibility and safety of combining catheter ablation and left atrial appendage closure in a single procedure. Chin Med J (Engl).

[20] Eckel RH, Cornier MA (2014). Update on the NCEP ATP-III emerging cardiometabolic risk factors. BMC Med.

[21] Tzikas A, Holmes DR, Gafoor S, Ruiz CE, Blomström-Lundqvist C, Diener HC, Cappato R, Kar S, Lee RJ, Byrne RA (2017). Percutaneous left atrial appendage occlusion: the Munich consensus document on definitions, endpoints, and data collection requirements for clinical studies. Ep Europace.

[22] Kar S, Hou D, Jones R, Werner D, Swanson L, Tischler B, Stein K, Huibregtse B, Ladich E, Kutys R (2014). Impact of Watchman and Amplatzer devices on left atrial appendage adjacent structures and healing response in a canine model. JACC Cardiovasc Interv.

[23] Massarenti L, Yilmaz A (2012). Incomplete endothelialization of left atrial appendage occlusion device 10 months after implantation. J Cardiovasc Electrophysiol.

[24] McIvor F, Wall D (2019). Who watches the WATCHMAN? A case of incomplete endothelialization at 3 years after device implantation. Eur J Cardiothorac Surg.

[25] Viles-Gonzalez JF, Kar S, Douglas P, Dukkipati S, Feldman T, Horton R, Holmes D, Reddy VY (2012). The clinical impact of incomplete left atrial appendage closure with the Watchman Device in patients with atrial fibrillation: a PROTECT AF (Percutaneous Closure of the Left Atrial Appendage Versus Warfarin Therapy for Prevention of Stroke in Patients With Atrial Fibrillation) substudy. J Am Coll Cardiol.

[26] Saw J, Lopes JP, Reisman M, McLaughlin P, Nicolau S, Bezerra HG (2016). Cardiac computed tomography angiography for left atrial appendage closure. Can J Cardiol.

[27] Tang X, Zhang Z, Wang F, Bai Y, Xu X, Huang X, Zhao X, Gong S, Qin Y (2017). Percutaneous left atrial appendage closure with LACBES(®) Occluder—a preclinical feasibility study. Circ J.

[28] Wang Z, Wang Y, Ye J, Lu X, Cheng Y, Xiang L, Chen L, Feng W, Shi H, Yu X (2015). bFGF attenuates endoplasmic reticulum stress and mitochondrial injury on myocardial ischaemia/reperfusion via activation of PI3K/Akt/ERK1/2 pathway. J Cell Mol Med.

[29] Dong X, He Z, Xiang G, Cai L, Xu Z, Mao C, Feng Y (2020). Paeoniflorin promotes angiogenesis and tissue regeneration in a full-thickness cutaneous wound model through the PI3K/AKT pathway. J Cell Physiol.

[30] Zhang W, Bai X, Zhao B, Li Y, Zhang Y, Li Z, Wang X, Luo L, Han F, Zhang J (2018). Cell-free therapy based on adipose tissue stem cell-derived exosomes promotes wound healing via the PI3K/Akt signaling pathway. Exp Cell Res.

[31] Dukkipati SR, Kar S, Holmes DR, Doshi SK, Swarup V, Gibson DN, Maini B, Gordon NT, Main ML, Reddy VY (2018). Device-Related Thrombus after left atrial appendage closure: incidence, predictors, and outcomes. Circulation.

[32] Saw J, Nielsen-Kudsk JE, Bergmann M, Daniels MJ, Tzikas A, Reisman M, Rana BS (2019). Antithrombotic therapy and device-related thrombosis following endovascular left atrial appendage closure. JACC Cardiovasc Interv.

[33] Saw J, Tzikas A, Shakir S, Gafoor S, Omran H, Nielsen-Kudsk JE, Kefer J, Aminian A, Berti S, Santoro G (2017). Incidence and clinical impact of device-associated thrombus and peri-device leak following left atrial appendage closure with the amplatzer cardiac plug. JACC Cardiovasc Interv.

[34] Reddy VY, Doshi SK, Kar S, Gibson DN, Price MJ, Huber K, Horton RP, Buchbinder M, Neuzil P, Gordon NT (2017). 5-Year outcomes after left atrial appendage closure: from the PREVAIL and PROTECT AF Trials. J Am Coll Cardiol.

